# Motion-induced blindness shows spatial anisotropies in conscious perception

**DOI:** 10.1038/s41598-024-78939-6

**Published:** 2024-11-12

**Authors:** András Sárközy, Jonathan E. Robinson, Gyula Kovács

**Affiliations:** 1 Friedrich Schiller University, 07743 Jena, Germany; 2 Monash Centre for Consciousness and Contemplative Studies, Monash University, Clayton, 3168 Australia

**Keywords:** MIB, Motion-induced blindness, Visual field anisotropy, Polar angle asymmetry, HVA, Eye tracking, Consciousness, Attention, Perception, Object vision, Human behaviour

## Abstract

Polar angle asymmetries (PAAs), the differences in perceptual experiences and performance across different regions of the visual field are present in various paradigms and tasks of visual perception. Currently, research in this area is sparse, particularly regarding the influence of PAAs during perceptual illusions, highlighting a gap in visual cognition studies. We aim to fill this gap by measuring PAAs across the visual field during an illusion applied to test conscious vision widely. Motion-induced blindness (MIB) is an illusion when a peripheral target disappears from consciousness as the result of a continuously moving background pattern. During MIB we separately measured the average disappearance time of peripheral targets in eight equidistant visual field positions. Our results indicate a significant variation in MIB disappearance times and frequencies as a function of target location. Specifically, we found shorter and fewer disappearances along the cardinal compared to oblique directions, and along the horizontal compared to the vertical meridian. Our results suggest specific consistencies between visual field asymmetries and conscious visual perception.

## Introduction

The organization of the visual system has been studied over the years extensively, revealing the systematic spatial mapping of the visual field onto different cortical areas. One crucial aspect of this mapping involves the eccentricity of the location of a stimulus, referring to its distance to a central fixation point. Eccentricity affects visual acuity greatly: best at the fovea and decreases with eccentricity. This phenomenon is well-supported by the density gradient of cones peaking at the fovea and declining towards the periphery^1–3^. In addition, at a given eccentricity, the location or polar angle of the position of a stimulus in relation to the fixation center influences visual performance as well. First, visual performance is better along the horizontal and vertical meridians compared to oblique orientations^4^. Second, several studies have identified asymmetries, labeled as horizontal-vertical anisotropy (HVA), in the sense that performance in orientation discrimination, detection, and localization tasks along the horizontal meridian is superior to that over the vertical one^5–7^. Furthermore, it has been shown that the visual performance is better, at least in adult participants (for the lack of effect in children and adolescents, see Carrasco et al.^8^), for stimuli over the lower when compared to the upper part of the vertical meridian^9^, a phenomenon called vertical meridian asymmetry (VMA). The neural correlates of these polar angle asymmetries (PAAs) proved to be mainly rooted in the distribution of photoreceptors and midget retinal ganglion cells in human participants, which show higher densities along the horizontal when compared to the vertical meridian, and along the lower compared to the upper vertical meridian^10–12^. The visual field representation in the macaque’s primary visual cortex (V1) shows also HVA as the largest surface area is allocated to the horizontal meridian^13^. This asymmetry has been subsequently proven in human studies as well^14,15^. Such retinal and cortical asymmetries may account for the observed psychophysical HVA effects^16,17^ and can be observed for example in contrast sensitivity, orientation discrimination, and acuity^5,18,19^, and it has also been demonstrated in short-term memory experiments^20^. While PAAs are well-documented phenomena^21^ and seem to be shown in a large variety of stimuli and tasks, they remain unexplored in optical illusions, which allow us to delineate the contribution of internal states to PAAs from the early visual asymmetries described above.

We used Motion-induced blindness (MIB)^22^ for our study of PAAs in conscious perception as this illusion involves higher-level visual processes closely linked to conscious awareness. Unlike several other illusions that primarily result from early visual processing, for example, Hering’s illusion or the Muller-Lyer illusion^23,24^, MIB is well-suited for investigating phenomena related to the dynamic interaction between conscious and unconscious visual inputs. Specifically, MIB entails the suppression of peripheral stimuli from awareness due to the salience of a moving background, which aligns with higher-order cognitive mechanisms. For instance, MIB was recently modeled as a noisy excitable system^25^, where the duration of the illusion reflects the visual system’s efficiency in restoring conscious awareness. Thus, MIB provides a unique opportunity to explore PAAs as they relate to conscious perception, differentiating it from early-stage visual field asymmetries. Beyond its application in psychophysical experiments, its importance is highlighted by using it in real-life simulations^26,27^. In the context of MIB, the brain appears to prioritize the more salient visual information (in this case the motion of the background pattern), leading to the disappearance of the peripheral stationary target from awareness^22^. By investigating polar angle asymmetries within the MIB illusion, we differentiate perceptual biases, which we refer to as perceptual performance, from early visual asymmetries. In the current work, we test if the MIB illusion shows any PAAs. We tested the magnitude of MIB in eight peripheral positions, equidistant from the fixation center, spanning the entire 360-degree visual field. We hypothesized that shorter disappearance times in the MIB illusion would correlate with better perceptual performance. Specifically, we expected that MIB would exhibit a smaller magnitude (shorter disappearance times and lower frequency of disappearances): (1) along the horizontal compared to the vertical meridian leading or contributing to HVA; (2) in the lower part compared to the upper part of the vertical meridian, according to VMA, and (3) along cardinal directions compared to oblique directions. We aim to investigate whether the dynamics observed in the MIB illusion vary across the visual field in a manner consistent with early-stage visual anisotropies. If our hypothesis holds, this could suggest that these anisotropies are not solely a product of early visual processing but are also shaped by higher-level mechanisms involved in conscious perception.

## Results

Before analysis, all position and visual field area data were evaluated using Q-Q plots to confirm adherence to a normal distribution, ensuring the validity of our statistical test.

**Fig. 1:**
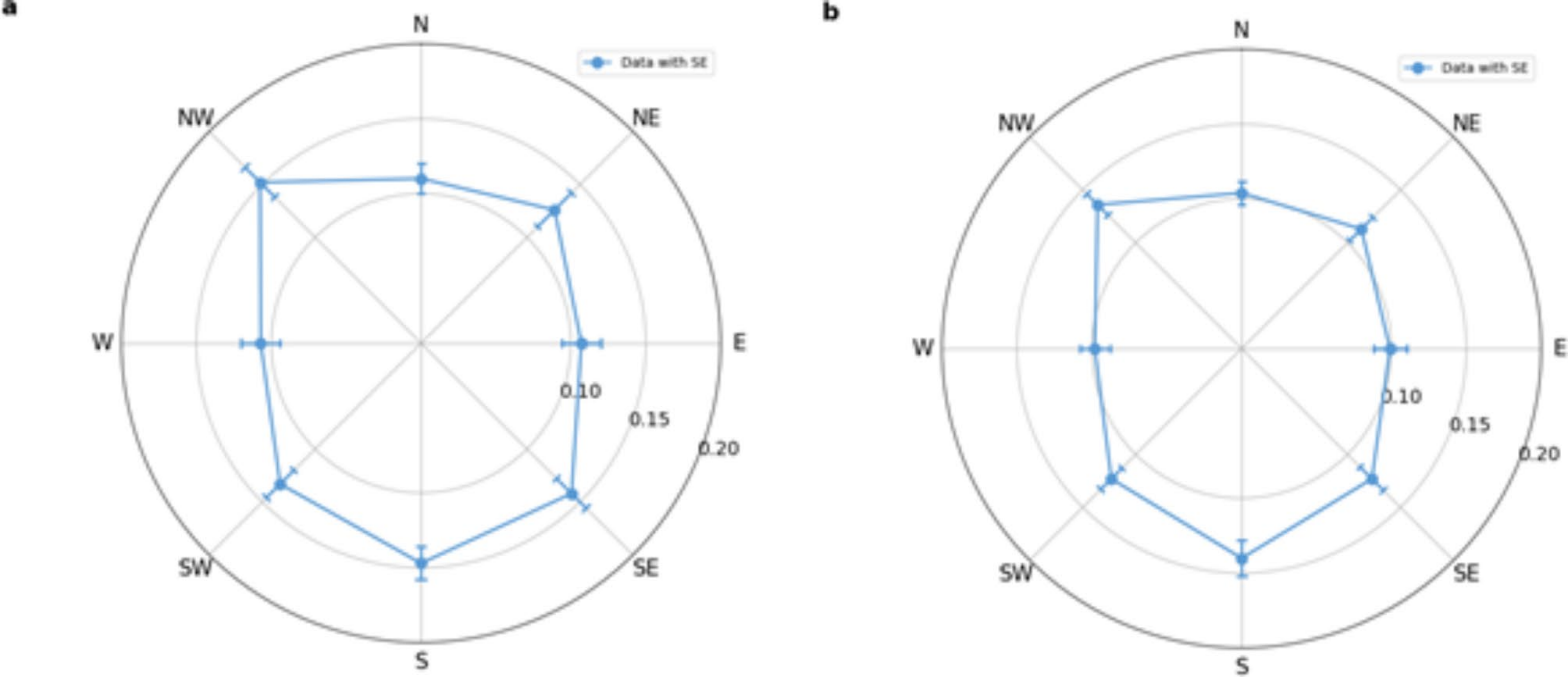
Mean total times (**a**) and frequencies (**b**) of the MIB illusion for each position separately (+-SE). The polar plots show significant differences in disappearance times and frequencies across the visual field positions. N-north, NE-northeast, E-east, SE-southeast, S-south, SW-southwest, W-west, NW-northwest.

Applying the Greenhouse-Geisser correction due to the violation of the sphericity assumption, the length of the MIB illusion was significantly modulated by the position of the peripheral target (F(5.159, 123.822) = 5.478, *p* < 0.001, η^2^ = 0.186), indicating a strong anisotropy across the visual field. Furthermore, we also examined the frequency (the time-normalized number of disappearances) for additional robustness, which confirmed the observed effect (F(4.552, 109.259) = 6.582, *p* < 0.001, η^2^= 0.215), providing further evidence for the spatial modulation of the illusion (Table 1; Fig. 1).

**Table 1:** The time-normalized disappearance time and frequency means(+-SE) measured, pro position.

	Positions							
	E	*N*	NE	NW	S	SE	SW	W
Normalized sum of disappearance time [%]	0.107	0.11	0.126	0.152	0.147	0.142	0.133	0.107
**SE**	0.013	0.01	0.016	0.014	0.011	0.014	0.013	0.013
Normalized frequency of disappearance [Hz]	0.099	0.104	0.113	0.136	0.140	0.123	0.123	0.098
**SE**	0.011	0.008	0.011	0.01	0.012	0.011	0.01	0.01

Post hoc analyses, employing Bonferroni correction, revealed significant differences among the following visual field positions for the length of illusion (Fig. 1a). Periods when participants reported the disappearance of the peripheral targets were significantly shorter in the East position when compared to the Northwest (t(24)=-4.031, *p* = 0.002, *d*=-0.685), South (t(24)=-3.584, *p* = 0.012, *d*=-0.609), and Southeast position (t(24)=-3.196, *p* = 0.047, *d*=-0.543). The analysis of the frequency of the disappearances supported these findings (Fig. 1b), showing a similarly reduced frequency of the MIB illusion in the East position, when compared to the Northwest (t(24)=-4.168, *p* = 0.001, *d*=-0.708) and South(t(24)=-4.603, *p* < 0.001, *d*=-0.782) positions.

Also, targets at the West position (M = 0.107, SD = 0.065) led to significantly shorter periods of disappearances when compared to the Northwest (t(24)=-4.037, *p* = 0.002, *d*=-0.686), South (t(24)=-3.59, *p* = 0.012, *d*=-0.61), and the Southeast position (t(24)=-3.202, *p* = 0.046, *d*=-0.544).

West position also showed lower frequency of disappearance compared to Northwest (t(24)=-4.268, *p* < 0.001, *d*=-0.725) and South (t(24)=-4.704, *p* < 0.001, *d*=-0.799) positions.

Targets at the Northwest position were associated with significantly longer periods of disappearances than targets at the North position (t(24) = 3.741, *p* = 0.007, *d* = 0.636), additionally South position showed significantly longer disappearances than the North (t(24) = 3.294, *p* = 0.034, *d* = 0.56). Similarly, comparing the disappearance frequencies, Northwest position here exhibited a significantly higher frequency compared to the North position (t(24) = 3.631, *p* = 0.009, *d* = 0.617), and the South position showed a higher number of disappearances than the North (t(24) = 4.066, *p* = 0.002, *d* = 0.691) position. No other differences were significant.

**Fig. 2:**
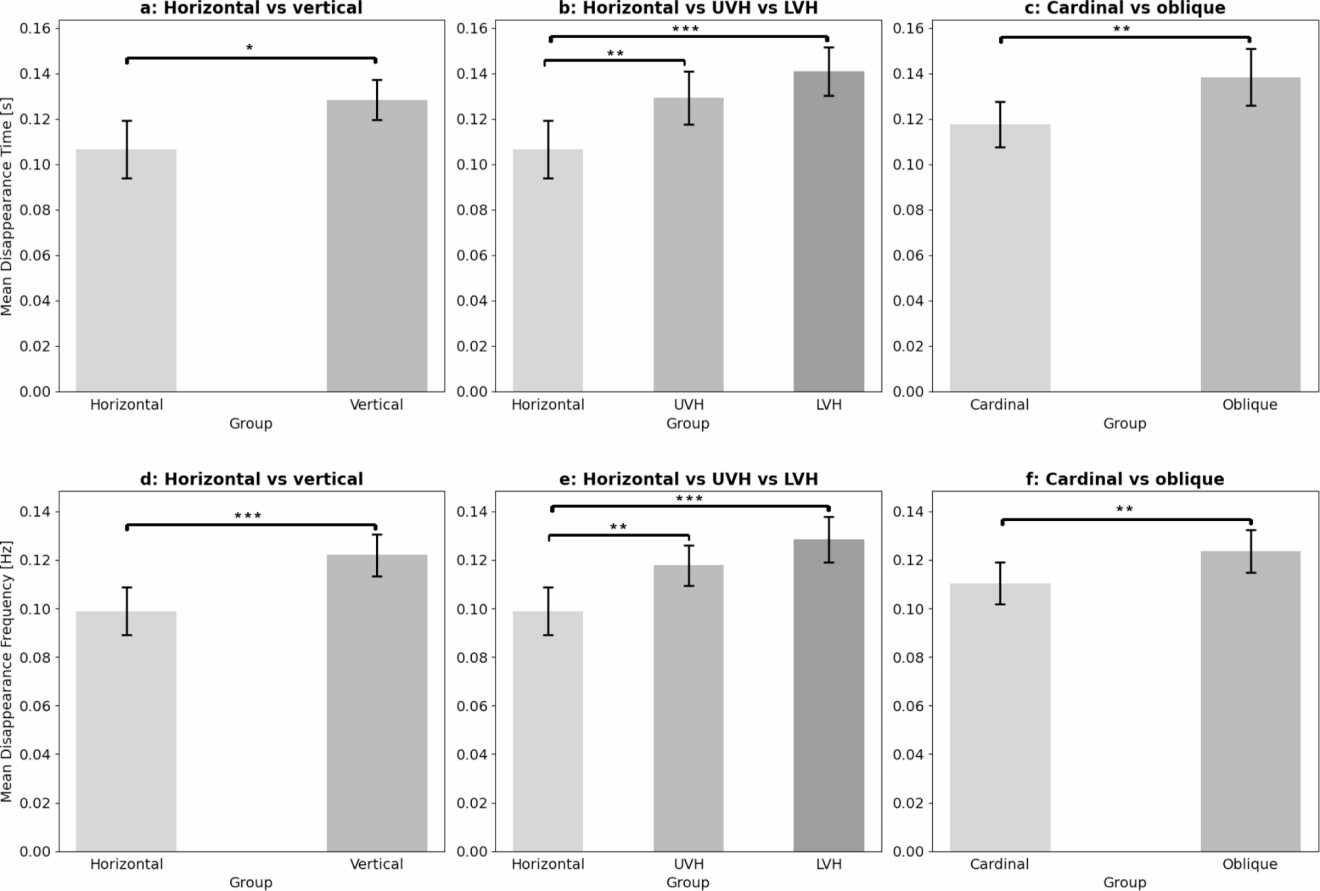
Mean normalized times (+-SE) of the MIB illusion for positions grouped across the meridians (**a**), the horizontal meridian vs. the UVH and LVH (**b**), the cardinal and oblique axes (**c**), and frequencies (+-SE) in the same order (**d**, **e**, **f**). For definitions of the groups see Table 2.

Previous research has suggested anisotropies across the visual field, specifically between the vertical and horizontal meridians^18^, motivating our explicit test of this phenomenon for MIB as well. The Paired Sample t-tests revealed significantly shorter mean disappearance times (Fig. 2a*)* for the horizontal when compared to the vertical meridian (t(24)=-2.688, *p* = 0.013, *d*=-0.538). To further support our findings on HVA, we also implemented a Paired Sample t-test on the number of disappearances between the horizontal and vertical meridian, where we found a significant difference ((t(24)=-3.750, *p* < 0.001, *d*=-0.750) again (Fig. 2d).

We aimed to investigate whether differences in the times of disappearance exist not only between the horizontal and vertical meridians but also across other areas of the visual field. To achieve this, we grouped peripheral target locations into UVH and LVH (Table 2) and compared the mean disappearance times of those with the horizontal meridian (Fig. 2b). We performed a Repeated Measures ANOVA with *position* (3 - UVH, LVH, and horizontal meridian) as within-subject factor and observed a statistically significant main effect (F(2, 48) = 12.322, *p* < 0.001, partial η²=0.339). Targets located at the horizontal meridian led to significantly shorter disappearance times when compared to both the UVH area (t(24)=-3.208, *p* = 0.007 *d*=-0.384) as well as the LVH area (t(24)=-4.885, *p* < 0.001, *d*=-0.584), employing post hoc analysis with Bonferroni correction. We applied the same analysis to the frequency of disappearances to reinforce the distinctiveness of the horizontal meridian. A Repeated Measures ANOVA here again showed a robust main effect (F(2, 48) = 14.629, *p* < 0.001, partial η²=0.379). The Bonferroni post hoc test revealed significantly fewer disappearances on the horizontal meridian compared to the UVH area (t(24)=-3.419, *p* = 0.003, *d*=-409) and to the LVH area (t(24)=-5.339, *p* < 0.001, *d*=-0.639) as well (Fig. 2e*).*

Finally, we tested if positions along the cardinal (horizontal and vertical) meridians led to different MIB disappearance times when compared to positions along the two oblique axes (NW-SE and NE-SW). For this analysis, we averaged the disappearance times, obtained over positions along the horizontal and vertical meridians as well as over those of the two oblique axes (Table 2; Fig. 2c and f). A Paired Sample t-test demonstrated significantly shorter disappearance times for the cardinal when compared to the oblique angles (t(24)=-3.205, *p* = 0.004, *d*=-0.641). For robustness, we also implemented the analysis here to the frequency of disappearances, finding significantly fewer disappearances along the cardinal axes compared to those oblique ones ((t(24)=-3.560, *p* = 0.002, *d*=-0.712).

Overall, these results suggest a strong anisotropy across the visual field for the length of time and the frequency of disappearances in the MIB illusion.

## Discussion

We explored the polar angle asymmetries – measuring perceptual performance through the duration of illusion experiences in MIB. We hypothesized that the MIB illusion exhibits significant variations across the visual field, suggesting variations in perceptual performance. A shorter duration of the illusion reflects the visual system’s efficiency in reestablishing conscious awareness of the target, which one can, in turn, interpret as a sign of stronger perceptual and/or attentional processes. While early stages of visual processing (such as sensitivity to contrast or adaptation to the moving inducer pattern) may contribute to the MIB effect, the recovery from perceptual disappearance is likely governed by higher-level mechanisms. For instance, MIB can be modeled as an excitable system^25^, where spontaneous fluctuations in neural activity cause the disappearance and subsequent recovery of the target. In this framework, a weaker MIB effect, indicated by shorter disappearance times, signifies that the brain is better able to counteract the disruptive influence of the moving background when compared to cases with stronger MIB illusion, thereby restoring awareness of the target with greater efficiency. Consistent with previous studies, our findings show that participants experience the illusion for shorter durations and observe less number of disappearances for positions along cardinal directions (N, E, S, W) compared to oblique positions (NE, SE, SW, NW). Visual meridians generally show a higher density of photoreceptors, midget retinal ganglion cells, and also an excessive amount of surface area in the V1 ^21^ which can explain the weaker performance in the obliques, inducing longer duration of illusion in these positions. This asymmetry has also been observed earlier in orientation discrimination line grating tasks^28^, revealing maximal acuity when the gratings were oriented horizontally or vertically. This phenomenon, wherein visual performance is superior in horizontal and vertical (cardinal), as opposed to oblique orientations, is commonly known as the “oblique effect”^29^.

Attentional mechanisms may act to amplify the perceptual differences between cardinal and oblique orientations observed in our study. Bloem and Ling^30^ demonstrated that attentional load disproportionately impacts oblique orientations, where reduced attentional resources lead to greater declines in perceptual sensitivity compared to cardinal orientations. This aligns with the “response gain” mechanism, where cardinal orientations, with their broader neural representation, are more resistant to perceptual suppression under constrained attention. Applying this to our findings on motion-induced blindness (MIB), the shorter disappearance times and lower disappearance frequencies for cardinal orientations may reflect their advantage in engaging attentional resources. Thus, the anisotropies we observed may involve not only early visual processing but also attentional modulation, with cardinal orientations benefiting more from available attentional resources. Future studies could directly test this by varying attentional load during MIB, providing a clearer picture of how attentional dynamics influence visual field anisotropies in conscious perception.

Our results also support that the well-established HVA phenomenon is present in visual illusion as well, manifesting in shorter lengths of illusion (and less frequent disappearances) along the horizontal compared to the vertical meridian. These findings are in accord with previous studies showing HVA in the traveled distance estimation paradigm, 3D exocentric pointing task, and short-term memory tasks^6,7,20^. This highlights the fact that asymmetries around visual field meridians show a robust correlation with visual illusion experiences as well.

There is a rich body of literature on the differences between right and left visual hemifield, as well as the UVF and LVF, depending on the nature of the experimental tasks^31,32^. In the context of MIB, we did not find any significant differences between these visual areas. Interestingly, we found the opposite results – although, only when we compared the N and S points to each other, - regarding the VMA. This result diverges from some – however not very robust – prior findings in spatial resolution^33^ and orientation discrimination^9^ tasks. The absence of VMA in the current study could potentially be attributable to our sample’s age group (M = 21.64, SD = 2.72). Recent work^8^ suggests that visual perception – in the case of the VMA - develops beyond childhood, with possible causes of late-stage changes – environmental influences, inherent development patterns, or both. Results opposite to the VMA have mostly been found in visual search tasks so far^34^, so the origin of this discrepancy in MIB is currently unclear and requires further investigation.

Our research also quantified prior observations where participants observed the most frequent disappearances in MIB in the upper left quadrant of the visual field, allegedly due to lower perceptual performance in this area ^22,35^. This is contradictory to studies where stimuli in the left hemifield led to superior performance in various tasks such as line bisection^36^ and the landmark task^37^. These studies suggest a natural attentional bias to the left, akin to ‘pseudoneglect’, mirroring the disturbances seen in hemispatial neglect patients^38^. However, we did not find this upper left difference in our work.

Explanation of longer disappearances in the upper left corner, when multiple targets are presented simultaneously, might come from predictive coding models, which propose that the brain constantly predicts sensory input and actively endeavors to minimize the difference between expected and actual signals^39^. This upper left visual field anomaly could be due to an increased precision weighting of evidence in this area. Under this explanation, the increased precision on imprecise (low-quality) evidence from the periphery would lead to an increased disappearance as the grid pattern dominates the space sampled in the periphery. To speculate on this particular upper-left effect this may be attributable to habitual reading patterns (left to right in many languages) and/or other culturally driven visual habits (e.g. menu bars of computer operating systems), leading to an altered processing strategy for illusions as well. Watanabe et al.^40^ explored how predictive coding can account for generating motion illusions. By training a predictive coding-based neural network the model was able to predict both real and illusory motion, supporting the idea that mechanisms assumed by predictive coding theory form one basis of motion illusion generation. This finding could be pertinent in understanding why this specific area may exhibit heightened illusion rates under the MIB paradigm. Furthermore, Carter et al.^41^ examined how the number of objects, their grouping, and observer attention affect object disappearance in MIB. Experiments showed that increases in object count and specific groupings slightly raised disappearance rates, but significantly, enhanced attention to objects markedly increased their chance of vanishing. These findings support the conclusion that while object arrangement has a modest impact, it is the allocation of attention that crucially influences perceptual visibility, leading to a paradoxical increase in object disappearance with greater attention. Similar results in MIB demonstrate^42^ the role of attention in visual perception, where directly focusing on a chosen object (out of two) can paradoxically make it more likely to disappear from perception. Attention might amplify certain neural mechanisms that lead to the disappearance of the target, such as perceptual filling-in, where our brain fills in the space where the target disappears with the surrounding background^43^ or disruption of attentional switching between target and distractors. Nevertheless, these findings were not fully explained, within the scope of our findings, the heightened covert attention to the upper left region (a prime area for reading and computer use, as discussed before) might lead to a higher frequency of disappearances.

Future neuroimaging studies could provide insights into the cortical areas involved in the differences in perceptual performance, specifically in illusions. Exploring whether similar disparities are present in other visual illusions, such as the “double flash illusion”, is of interest to determine if the asymmetrical findings observed in MIB persist. If asymmetries do not emerge, it would be instructive to investigate the divergent characteristics of these illusions. Furthermore, our analysis did not consider the potential influence of learning or adaptation effects on the duration of illusion experiences. It is possible that repeated exposure to the MIB task could alter perceptual performance, either enhancing or reducing the duration of illusions over time. Longitudinal studies could address this by examining changes in performance across multiple sessions. Another consideration is the ecological validity of our findings. The controlled laboratory setting in which the MIB illusion is experienced may not accurately reflect visual performance in more naturalistic environments. Future research could look to replicate these findings in settings that more closely mirror everyday visual tasks. Earlier study^44^ showed that the contrast dependency of our two measures of MIB magnitude (length of illusion periods and disappearance rate of the target stimulus) is different: in the sense that enhancing contrast decreased disappearance time but increased the rate of disappearances, suggesting distinct underlying mechanisms behind the two. Unlike in the mentioned work, in our study, the two applied measures led to identical results regarding PAAs. This suggests that the contrast and position dependence of MIB rely on different background mechanisms.

In addition, we also propose several avenues for future research. One particularly promising area is the exploration of the role of attentional networks in the MIB phenomenon. While our study hints at the involvement of attentional mechanisms, subsequent research could employ tasks specifically designed to tease apart the contributions of attentional networks to the experience of visual illusions. The development of computational models that simulate visual processing in the human brain could also advance our understanding of visual asymmetries in visual illusions. Such models could be used to predict individual differences in illusion experiences and potentially inform the design of interventions to enhance visual/perceptual performance.

Another interesting line of experiments could compare the PAAs of bistable stimulations, such as binocular rivalry (BR) with that of MIB. BR is thought to share computational principles with MIB^45^. The overlap of the neural processing of BR and MIB, however, is currently under debate. While some suggest identical oscillators behind the two processes^46^, others emphasize the different nature of them^47^. To the best of our knowledge, no study has yet tested the PAAs of BR, therefore its comparison to that of MIB would also be a direction for future experiments.

Based on our study, the biases observed in perceptual performance during MIB generally align with those in early visual as well as attentional processes. However, the subtle differences in MIB suggest that while perceptual biases are predominantly shaped by the quality of visual inputs, internal states also interact with these inputs to produce localized biases.

## Conclusions

We measured the MIB illusion across the entire visual field and found that its magnitude shows strong polar angle asymmetries for peripheral targets. We observed the shortest and least disappearance times/periods at positions over the horizontal meridian. Our results suggest that known asymmetries of the visual field extend to the level of conscious visual stimulus processing.

## Materials and methods

### Participants

A total of 30 participants (7 males), took part in the experiment, recruited by an internal system of the Insititute of Psychology, Friedrich Schiller University (Jena, Germany). All participants had normal (or corrected-to-normal) vision and showed no neurological or psychiatric symptoms. Additionally, participants were required to exhibit proficiency in computer literacy and sustain a steady fixation for a minimum of 5 s during the pre-test period. Participants who failed to report any disappearances of the target stimulus in at least 50% of the trials were excluded from the analysis (*N* = 3). One participant did not follow instructions while the data of another one was lost, due to software failure. Therefore the current analysis is based on 25 participants (5 males), falling within the age range of 18 to 30 years (M = 21.64, SD = 2.72). A post hoc power analysis, using G*power 3.1 ^48^ for within-subject ANOVA with 8 repeated measures factors showed that the achieved effect size of η^2^ = 0.186 with the obtained sample size leads to a high-power (1), suggesting that the sample size was adequate. Participants were rewarded by partial course credits. The study was approved by the local ethics committee (ethics number: FSV 21/073) and was conducted in accordance with the guidelines of the Declaration of Helsinki. Informed consent was obtained from all participants prior to their participation in the experiment.

#### Apparatus

**Fig. 3:**
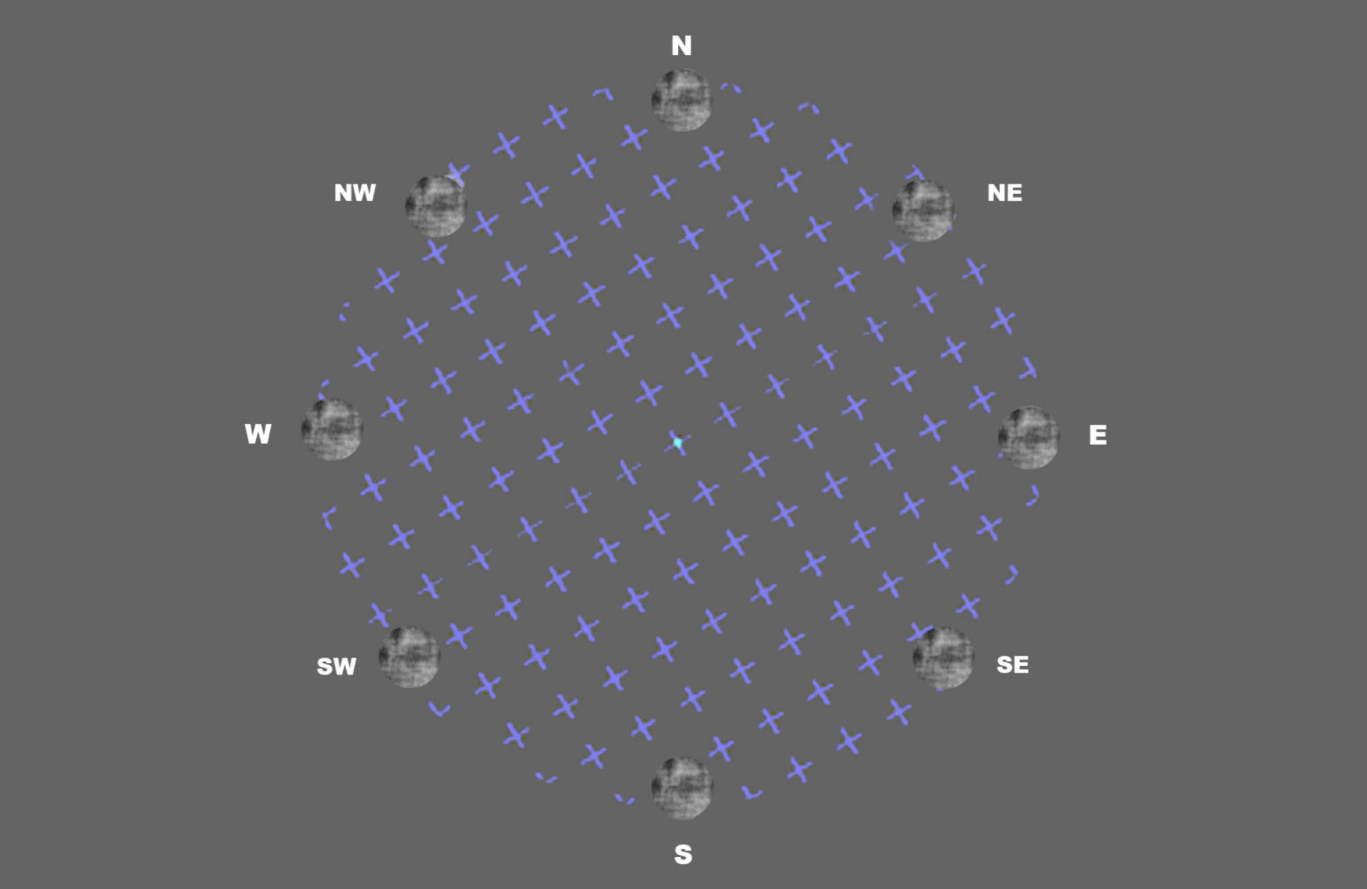
Illustration of the target stimulus and the MIB inducer pattern. The eight potential locations of the target stimulus are illustrated. Only one of these positions was presented at a time, randomly, per trial. The MIB inducer pattern of crosses rotated randomly in either a clockwise or counterclockwise direction. For abbreviations see Fig. 1. For details see Apparatus section.

The experiment was running on a Windows 11 Pro computer, using a Python 3.6 version to conduct the experiment. The experiment monitor, A DELL 2721DGFA was set to 2560 × 1440 resolution and 165.08 Hz refresh rate. Another monitor was connected to the same computer, running and supervising the experiment. An Eyelink 1000Plus (SR Research, Ottawa, Canada) eye tracker has been used for monitoring eye movement. The stimulus image was created using the SHINE toolbox^49^. The target stimulus was a circular patch (radius: 1.8528 deg), filled with a random pattern, created by the Fourier phase-randomization of one picture of the Racially Diverse affective expression (RADIATE) face stimulus set^50^. The image was matched in contrast and luminance to the background. The target stimulus was systematically presented at eight pre-defined peripheral locations, at the following polar angles (with ± 2 degree randomized variance): 0: North (N), 45: Northeast (NE), 90: East (E), 135: Southeast (SE), 180: South (S), 225: Southwest (SW), 270: West (W) and 315: Northwest (NW). Each position was at a distance of 8 degrees of visual angle from the central fixation point, with the position denoting the center of the target stimulus. For an illustration of the stimulus, see Fig. 3. The fixation dot was a centrally presented blinking dot alternately changing its color (white and blue) in 0.5-second intervals^22^. The MIB inducer pattern was a circle, filled with crosses (size: 0.22 deg; color: R: 78.32, G: 78.32, B: 254.63; inter-element distance: 0.22 deg, average luminance with background: 100.33 cd/m^2^), masked with a radius of 9 degrees visual angle from the central fixation dot. This pattern rotated at a rate of 0.37 cycles per second, matching that of Bonneh et al.^44^, either clockwise or counterclockwise, randomized across trials with equal probabilities.

### Experimental design

#### Fixation training

First, participants completed a brief fixation training^51^. During this phase participants’ eye movements were first calibrated and validated. They were instructed to maintain their fixation on the central fixation dot. Next, a peripheral target stimulus appeared on the screen, positioned randomly at polar angles of 45, 135, 225, and 315 (distance from the center: 9 degrees). Participants were instructed to make a saccadic eye movement to the location of the peripheral target and maintain fixation for a duration of at least 1 s. After each successful trial, participants were required to fixate the target 1 s longer, for a maximum of 5 s. This progressive goal-setting aimed to identify individuals who are unable to sustain fixation for extended periods. Throughout this training phase, participants received feedback if they failed to maintain fixation on the target for the required duration. A maximum of 20 training trials was presented and all participants completed the training phase successfully.

Subsequently, we conducted a demonstration to showcase perceptual disappearance, which involved the use of a physically removed stimulus. This demonstration aimed to help participants understand the distinct characteristics that are associated with the complete perceptual disappearance of targets. Finally, participants received detailed instructions (oral and written, on-screen) on the execution of the paradigm.

#### MIB paradigm

**Fig. 4:**
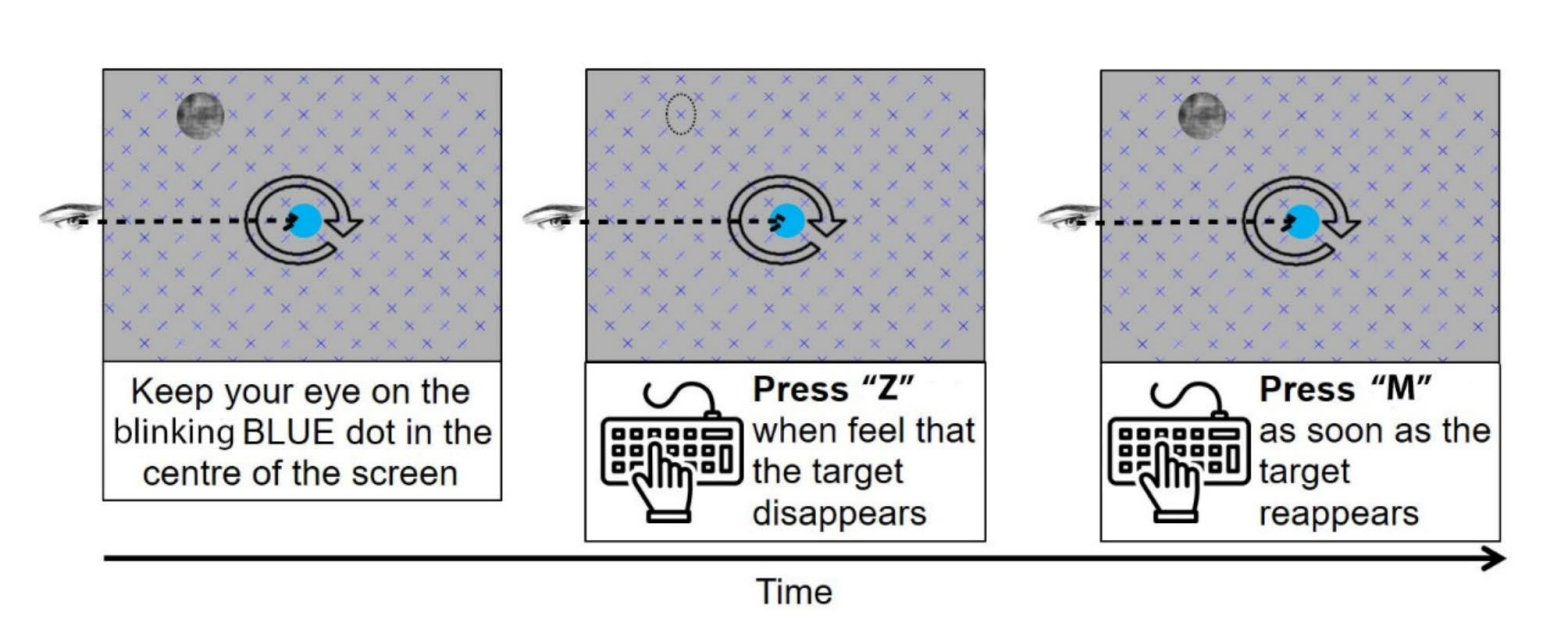
Storyboard of the experiment design, describing the task for the participants. Each screen was presented until a button was pressed at the beginning of each block of the experiment.

During the main experiment, 48 trials were present in 3 blocks, separated by self-timed breaks. One block contained 16 trials, repeating each of the 8 locations twice, with a randomized order. The duration of each trial was 45 s, enabling the participants to observe the disappearance of the target from consciousness multiple times. The entire experiment took approximately 36 min, excluding breaks. Participants were instructed to continuously fixate on the central fixation dot and maintain uninterrupted fixation for a minimum of 1.5 s before providing any responses regarding the disappearance of the peripheral stimulus. Trials were aborted when participants failed to maintain fixation. Participants’ task was to indicate the disappearance of the target stimulus from consciousness by pressing the Z key on a keyboard and the reappearance of the stimulus by pressing the M key (Fig. 4.

#### Analysis

To compare the length of the MIB illusion across positions, we subtracted the time of the button presses signaling target disappearances from those of the subsequent target reappearances, quantifying time „spent in illusion”. Next, these periods were summed for each position separately and normalized to the length of the sum of the trials, per position. This led to a number between 0 and 1; 0 indicates no illusion without any disappearance of the targets while 1 indicates the entire trial time „spent in illusion”. In addition, reports indicate that in bistable stimulations, such as binocular rivalry, the duration of the dominance of a perceptual state and the rate of state changes may lead to different results^52^, and increasing target contrast in MIB also could show similar effects^44^, thus we have also compared the frequency of MIB periods across positions. For this, we summarized the number of disappearances per position and normalized them in the same manner as we did for the duration times of illusion, relative to the total length of the trials for each position.

First, a simple 1-way ANOVA was performed to test the spatial specificity of MIB with position (8) as within-subject factor. Next, we divided the eight original positions into various visual field areas for further analysis. These areas included the horizontal and the vertical meridians, the upper visual hemifield (UVH), the lower visual hemifield (LVH), the cardinal, and the oblique axes (as shown in Table 2).

**Table 2:** The grouping of visual field positions was applied during the analysis.

Positions Included				Description
E	W			Horizontal meridian
N	S			Vertical meridian
N	NE	NW		Upper visual hemifield (UVH)
S	SE	SW		Lower visual hemifield (LVH)
S	N	E	W	Cardinal axes
SW	SE	NW	NE	Oblique axes

To test the HVA of MIB, we compared the horizontal and vertical meridians using a paired-sample t-test. Next, we compared the horizontal meridian to the UVH and LVH using a Repeated Measures ANOVA with position (3) as a within-subject factor. Finally, we compared the cardinal axes to the oblique ones, using a paired-sample t-test. For this analysis, the data of the two cardinal and two oblique axes were merged. To identify outlier trials we applied an “outer fence”^53^ criteria: F1 = q1 − 3 H-spread, F3 = q3 + 3 H-spread, where, q1 and q3 are the first and third quartiles, and H-spread equals the interquartile range (IQR). Excluded trials ranged from 0 to 3% for each participant’s data. Subsequent statistical analysis was performed by JASP (Version 0.18, *JASP Team*, 2023)^54^.

## Data Availability

The datasets generated during and/or analysed during the current study are available in a publicly accessible repository. The raw and processed data necessary to replicate and build upon the findings reported in this article have been deposited at OSF repository, available at https://osf.io/gcqem.
